# Assessing the optimal preparation strategy to minimize the variability of cardiac pyruvate dehydrogenase flux measurements with hyperpolarized MRS

**DOI:** 10.1002/nbm.3992

**Published:** 2018-07-24

**Authors:** Kerstin N. Timm, Andrew Apps, Jack J. Miller, Vicky Ball, Cher‐Rin Chong, Michael S. Dodd, Damian J. Tyler

**Affiliations:** ^1^ Department of Physiology, Anatomy and Genetics University of Oxford UK; ^2^ Oxford Centre for Clinical Magnetic Resonance Research, John Radcliffe Hospital Oxford UK; ^3^ Clarendon Laboratory, Department of Physics University of Oxford UK

**Keywords:** cellular and molecular cardiovascular imaging, diabetes, hyperpolarized ^13^C

## Abstract

Hyperpolarized [1‐^13^C] pyruvate MRS can measure cardiac pyruvate dehydrogenase (PDH) flux in vivo through ^13^C‐label incorporation into bicarbonate. Using this technology, substrate availability as well as pathology have been shown to modulate PDH flux. Clinical protocols attempt to standardize PDH flux with oral glucose loading prior to scanning, while rodents in preclinical studies are usually scanned in the fed state. We aimed to establish which strategy was optimal to maximize PDH flux and minimize its variability in both control and Type II diabetic rats, without affecting the pathological variation being assessed. We found similar variances in the bicarbonate to pyruvate ratio, reflecting PDH flux, in fed and fasted/glucose‐loaded animals, which showed no statistically significant differences. Furthermore, fasting/glucose loading did not alter the low PDH flux seen in Type II diabetic rats. Overall this suggests that preclinical cardiac hyperpolarized magnetic resonance studies could be performed either in the fed or in the fasted/glucose‐loaded state. Centres planning to start new clinical studies with cardiac hyperpolarized magnetic resonance in man may find it beneficial to run small proof‐of‐concept trials to determine whether metabolic standardizations by oral or intravenous glucose load are beneficial compared with scanning patients in the fed state.

**Abbreviations**
AUC: area under the curve
DNP: dynamic nuclear polarization
FA: fatty acid
GIK: glucose, insulin and potassium
HOMA‐IR: homeostatic model assessment of insulin resistance
IVGTT: intravenous glucose tolerance test
PDH: pyruvate dehydrogenase
STZ: streptozotocin
T2DM: Type II diabetes mellitus
TCA: tricarboxylic acid

## 1 INTRODUCTION

The heart catabolizes a mix of fatty acids (FAs), glucose, lactate and ketone bodies to meet its high energy demand.^1^ The relative contribution of each as a fuel source is determined by substrate availability (fed and fasted state), workload, oxygen supply and various pathologies.^2^ The healthy adult heart derives the majority (~70%) of its ATP from the β‐oxidation of FAs, with a shift to glucose oxidation after feeding.^3^ Carbohydrate and FA catabolism in the heart are thus reciprocally controlled and this inverse relationship is termed the Randle cycle.^4^ The pyruvate dehydrogenase (PDH) complex links glycolysis with the tricarboxylic acid (TCA) cycle and high levels of acetyl‐CoA derived from FA oxidation inhibit this multi‐enzyme complex both allosterically and via the activation of PDH kinases, which reversibly phosphorylate and inhibit PDH. PDH is dephosphorylated by PDH phosphatases, which are activated by calcium, by magnesium and by insulin signalling. This dephosphorylation and activation of PDH is impaired in in the diabetic heart.^5^ In the diabetic heart impaired insulin signalling furthermore leads to inhibition of PDH activity through upregulation of PDH kinases.^6^ Inactivation of PDH with continued flux through glycolysis controls the switch between aerobic and anaerobic glucose metabolism, which occurs, for example, in cardiac ischemia.^7^

Investigating cardiac metabolism in vivo is now possible via a number of evolving techniques.^8^ Dissolution dynamic nuclear polarization (DNP) can achieve hyperpolarization of ^13^C‐labelled substrates, which after injection into a living system allows real time assessment of downstream metabolic fluxes by MRS or MRI.^9^ ^13^C MRS of [1‐^13^C] pyruvate permits measurements of PDH flux via incorporation of the ^13^C label into bicarbonate. Recently, sterile hyperpolarizer systems have allowed the first in vivo studies of PDH flux in the resting human heart.^10^, ^11^ In preclinical models, hyperpolarized [1‐^13^C] pyruvate MRS has been performed in isolated perfused hearts^12^ and in vivo in mice,^13^ rats^14^ and pigs.^15^ In rat hearts in vivo hyperpolarized [1‐^13^C] pyruvate demonstrates the magnitude of the shift in PDH flux from the fed to fasted state following an overnight fast, reducing the ^13^C‐labelled bicarbonate signal by 74%.^14^ A similar shift in PDH flux using this technique was shown in mice.^13^ PDH flux measurements using hyperpolarized [1‐^13^C] pyruvate MRS have subsequently been show to correlate with ex vivo enzymatic activity measurements of PDH.^16^ In fasted rats, infusion of glucose, insulin and potassium (GIK) showed increased flux through PDH, which demonstrates that GIK infusions can improve glucose oxidation in the heart.^17^ Increased PDH flux due to GIK infusion could also be shown in healthy pigs.^18^ Furthermore, a study in pig hearts showed increased PDH flux due to angiotensin‐II‐induced increased cardiac afterload.^19^

In diabetes, raised circulating FAs increase the expression of PDH kinases,^20^ resulting in a reduction in PDH flux. This can again be characterized in vivo with hyperpolarized MRS, where PDH flux was shown to be reduced by up to 80% in Type II diabetic rats when compared with healthy control rats.^20^, ^21^ PDH flux was furthermore shown to be reduced in mice with reversible diabetes and no dyslipidaemia, again using hyperpolarized [1‐^13^C]pyruvate.^22^ Together, these studies show the potential of hyperpolarized [1‐^13^C] pyruvate to detect metabolic changes in the heart driven by changes in hormone levels and substrate availability in both physiological states and disease.

Preclinical studies assessing PDH flux using hyperpolarized cardiac MRS are generally performed in overnight fed rodents,^14^, ^20^, ^21^ whilst clinical studies to date have been undertaken following an oral glucose load after either normal eating^10^ or an overnight fast.^11^ However, which approach actually yields more robust, reproducible measurements is unknown. Therefore, in healthy rats, we aimed to compare the variance of PDH flux in overnight fed rats with that in rats receiving a standardized intravenous glucose injection following an overnight fast. Furthermore we wanted to assess whether metabolic standardization through glucose injection could affect the established pathophysiological blunting of PDH flux previously observed in the Type II diabetic heart.

## 2 EXPERIMENT

### 2.1 Animal handling

**Figure 1:**
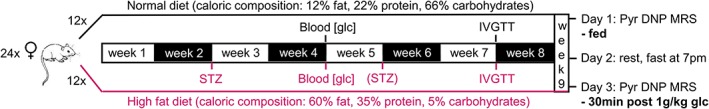
Study design. Female Wistar rats were maintained on either normal chow or a high fat diet for nine weeks. T2DM was induced by STZ injection and the diabetic phenotype characterized by fasting blood glucose levels at the start of Week 5 and an IVGTT at the start of Week 8. In Week 9 all rats underwent two hyperpolarized [1‐^13^C] pyruvate scans: one in the fed state and one after a rest day 30 min after an intravenous 1 g/kg glucose bolus following an overnight fast

All animal experiments conformed to the Animals (Scientific Procedures) Act, 1986, and were approved by a local ethics committee. The rats were housed in a 12:12 hour light/dark cycle in animal facilities at the University of Oxford. A model of Type II diabetes mellitus (T2DM) characterized by hyperglycaemia, hyperinsulinemia and hyperlipidaemia^23^ was induced based on a previously published protocol.^24^ Briefly, 24 age‐matched female Wistar rats (11 weeks of age at start of study) were divided into two groups: a control group receiving normal chow (12% calories from fat, 22% from protein and 66% from carbohydrates) and a T2DM group receiving a high fat diet for nine weeks (60% calories from fat, 35% from protein, 5% from carbohydrate). Two weeks after initiation of high fat feeding, rats in the T2DM cohort were injected intraperitoneally with a low dose (25 mg/kg) of freshly prepared streptozotocin (STZ) in citrate buffer (pH 4). Overnight fasting blood glucose was assessed by tail vein pricking under mild isoflurane anaesthesia (3 min at 2%) with an Accu‐Chek glucometer (Roche, Welwyn Garden City, UK) two weeks later in both cohorts. If blood glucose did not exceed 8 mM in the T2DM cohort, a second 25 mg/kg STZ injection was performed in Week 5. In Week 8, an intravenous glucose tolerance test (IVGTT)^25^ was performed to verify the T2DM phenotype and to assess insulin resistance. Rats were anaesthetized with 2% isoflurane in medical oxygen and a glucose solution was administered as a bolus (over 5 s) via a tail vein catheter with a dose of 1 g/kg at a concentration of 0.5 mg/mL in sterile water. Blood glucose levels were measured at time 0 (before glucose injection), 2, 5, 10, 15 and 30 min with an Accu‐Chek glucometer (saphenous vein‐prick), and blood samples for plasma insulin levels were taken at 0, 5, 15 and 30 min (~100 μL per sample into Li‐heparin‐coated tubes). Insulin levels were then quantified with an ELISA kit (Mercodia, Uppsala, Sweden) spectrophotometrically at 450 nm. The homeostatic model assessment of insulin resistance (HOMA‐IR) was calculated with the following equation:

$$\text{HOMA}-\mathrm{IR}=\frac{\left[\mathrm{glc}\right]\times \left[\text{insulin}\right]}{22.5\;\mathrm{mM}\times \mathrm{mU}/L}$$

where glucose concentration [glc] is given in mM and insulin concentration [insulin] in mU/L. Rats with a fasting glucose of 8‐14 mM were classified as T2DM and used for subsequent experiments. On the basis of these criteria all 12 rats in the control group were included in the study as well as nine of the 12 rats in the T2DM cohort. The overall study setup is illustrated in Figure 1.

### 2.2 Hyperpolarized [1‐^13^C] pyruvate measurements

In Week 9, rats received two hyperpolarized [1‐^13^C] pyruvate scans: one in the fed state, and one two days later, 30 min after a 1 g/kg glucose infusion (intravenous bolus over 5 s through a tail vein catheter) administered following an overnight fast (12‐18 h). The scans were performed between 7 am and 1 pm for both conditions. Rats were anaesthetized with 2% isoflurane in medical oxygen and placed in a 7 T Varian direct‐drive spectrometer (Santa Clara, CA, USA). Body temperature was maintained with a custom‐built handling system that also provided RF‐shielded ECG/RF interfaces.^26^ [1‐^13^C] pyruvic acid (Sigma‐Aldrich, Gillingham, UK) was hyperpolarized as described previously^27^ for 30 min in an alpha prototype hyperpolarizer (Oxford instruments, Abingdon, UK) at 3.35 T and 1.4 K. Dissolution was performed using a superheated alkaline solution, prepared with 2.4 g/L sodium hydroxide and 100 mg/L EDTA at 180 °C, yielding an 80 mM solution of hyperpolarized [1‐^13^C] pyruvate with physiological pH and temperature. Exactly 1 mL of 80 mM hyperpolarized pyruvate was injected into the tail vein over 10 s. ^13^C MR spectra were acquired in a 10 mm axial slice covering the heart, every second for 120 s using a 72 mm dual‐tuned birdcage volume transmit ^1^H/^13^C coil and a 40 mm ^13^C two‐channel surface receive coil (Rapid Biomedical, Rimpar, Germany) with a 15° gauss pulse and 13 kHz bandwidth. Multicoil spectra were recombined using an automatic whitened singular value decomposition method as previously described,^28^, ^29^ and 30 s of spectra, from the first appearance of the pyruvate resonance, were summed and quantified with AMARES/jMRUI.^30^ Blood glucose was measured before and 5 min after each hyperpolarized [1‐^13^C] pyruvate scan with an Accu‐Chek glucometer from a saphenous vein‐prick.

### 2.3 Statistical analysis

Unpaired and paired unequal variance Student *t*‐tests to analyse plasma glucose and insulin levels were performed in GraphPad Prism v6.01 (GraphPad, La Jolla, CA, USA) and significance was considered at *p* ≤ 0.05. Other statistical tests for the hyperpolarized MRS data were performed in R. Differences in means were assessed via a linear mixed effects model that represents a generalization of repeated measures ANOVA that is better placed to function in the presence of missing observations. The lme4 package was used in R with metabolic state and disease state considered as fixed effects, the subject ID considered as a random effect and the models fitted through maximum likelihood.^31^ Effect comparisons were made through a likelihood ratio test with lmerTest, and additionally the Akaike information criterion compared with models lacking the term of interest.^32^ Differences in variance were assessed via the non‐parametric Fligner‐Killeen test.^33^ Where applicable, Bonferroni's correction was used for multiple comparisons.

## 3 RESULTS

**Figure 2:**
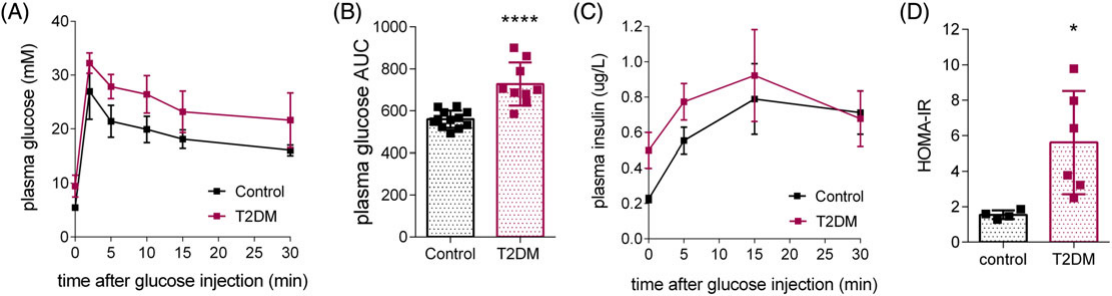
Diabetic model characterization. IVGTT in control (*n* = 12) and type II diabetic (T2DM, *n* = 9) rats. A, Blood glucose before (time 0) and at indicated times after an intravenous glucose bolus (1 g/kg). B, Total AUC of plasma glucose of the IVGTT in A. C, Plasma insulin levels measured before (time 0) and at indicated times after an intravenous glucose bolus (1 g/kg). D, HOMA‐IR in control (*n* = 4) and T2DM (*n* = 6) rats. Data are expressed as mean ± standard deviation. *p < 0.05, ****p < 0.0001

The T2DM rats showed increased fasting blood glucose levels and impaired glucose tolerance at all time points measured (2, 5, 10, 15 and 30 min after a 1 g/kg intravenous glucose injection) compared with their age‐matched controls (Figure 2A). The total plasma glucose area under the curve (AUC) was significantly higher in T2DM rats (727 ± 34) than in control rats (560 ± 12). However, T2DM rats showed no significant difference in plasma insulin levels in response to an intravenous glucose challenge at all time points measured (0, 5, 15 and 30 min) (Figure 2B). Fasting plasma glucose and insulin levels were very variable in the T2DM group, which resulted in a highly variable HOMA‐IR score (Figure 2C), reflecting different degrees of insulin resistance in the nine T2DM rats. The HOMA‐IR score was significantly higher in the T2DM rats compared with the control rats (*p* = 0.026), with the HOMA‐IR score being less than 2 for all animals in the control group and more than 2 for all animals in the T2DM group.

**Figure 3:**
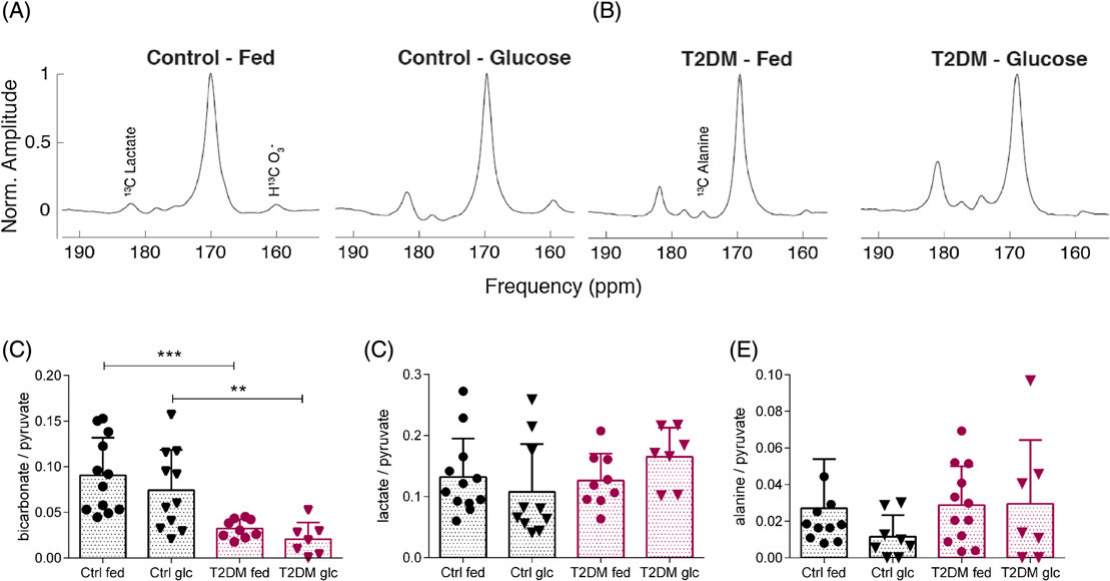
Hyperpolarized [1‐^13^C] pyruvate magnetic resonance spectroscopy in the heart. A,B, Carbon‐13 MR spectra from a 10 mm slab covering the heart of a representative control (A) and T2DM (B) rat both in the fed state and 30 min after an intravenous glucose bolus (1 g/kg) following an overnight fast. C‐E, Bicarbonate to pyruvate ratio (ctrl fed *n* = 12, ctrl glc *n* = 11, T2DM fed *n* = 9, T2DM glc *n* = 7) (C), lactate to pyruvate ratio (ctrl fed *n* = 12, ctrl glc *n* = 10, T2DM fed *n* = 9, T2DM glc *n* = 7) (D) and alanine to pyruvate ratio (ctrl fed *n* = 11, ctrl glc *n* = 8, T2DM fed *n* = 12, T2DM glc *n* = 7) (E) of the sum of the first 30 s of spectra from the first appearance of the pyruvate peak in fed and fasted/glucose‐loaded (glc) control (Ctrl) and T2DM rats. Data are expressed as mean ± standard deviation. **p < 0.01, ***p < 0.001

We compared the in vivo cardiac metabolism of [1‐^13^C] pyruvate in the T2DM model with control animals both in the fed state and 30 min after an intravenous glucose challenge delivered to a fasted animal (fasted/glucose loaded). Representative summed ^13^C spectra from a fed and fasted/glucose‐loaded control and a fed and fasted/glucose‐loaded T2DM rat heart are shown in Figure 3A,B, respectively. From summed spectra, ratios were taken of the peaks corresponding to bicarbonate, lactate and alanine to the pyruvate peak to normalize for any variation in polarization level between experiments (Figure 3C‐E).

In healthy control rats the bicarbonate to pyruvate ratio was 0.09 ± 0.01 in the fed state and 0.07 ± 0.01 in the fasted/glucose‐loaded state (Figure 3C). The lactate to pyruvate ratio was 0.13 ± 0.02 in the fed state and 0.11 ± 0.02 in the fasted/glucose‐loaded state (Figure 3D). Finally, the alanine to pyruvate ratio was 0.027 ± 0.008 in the fed state and 0.012 ± 0.004 in the fasted/glucose‐loaded state (Figure 3E). In the T2DM rats, the bicarbonate to pyruvate ratio was 0.032 ± 0.003 in the fed state and 0.021 ± 0.007 in the fasted/glucose‐loaded state (Figure 3C). The lactate to pyruvate ratio was 0.13 ± 0.01 in the fed state and 0.17 ± 0.02 in the fasted/glucose‐loaded state (Figure 3D). Finally, the alanine to pyruvate ratio was 0.029 ± 0.006 in the fed state and 0.03 ± 0.01 in the fasted/glucose‐loaded state. Overall, regardless of the disease state, PDH flux assessed by the bicarbonate to pyruvate ratio from hyperpolarized [1‐^13^C] pyruvate was not altered by the fasted/glucose‐loaded state compared with the fed state. Linear mixed modelling did not find any statistically significant differences in means between these groups for the bicarbonate to pyruvate ratio (*p* = 0.08), lactate to pyruvate ratio (*p* = 0.91) or alanine to pyruvate ratio (*p* = 0.99).

We hypothesized that the assessment of cardiac PDH flux in the fasted/glucose‐loaded state would yield reduced variability in the bicarbonate to pyruvate ratio due to standardization of substrate availability in the form of blood glucose, on the background of the standardized fasted state. However, regardless of disease state, there was no evidence to suggest that the variance in the data acquired was significantly reduced in the fasted/glucose‐loaded state compared with the fed state using the non‐parametric (unpaired) Fligner‐Killeen test for the homogeneity of variance (in the order bicarbonate to pyruvate ratio, lactate to pyruvate ratio, alanine to pyruvate ratio: *p* = 0.63, 0.90, 0.69 for the control group; *p* = 0.96, 0.75, 0.35 for the T2DM group).

**Figure 4:**
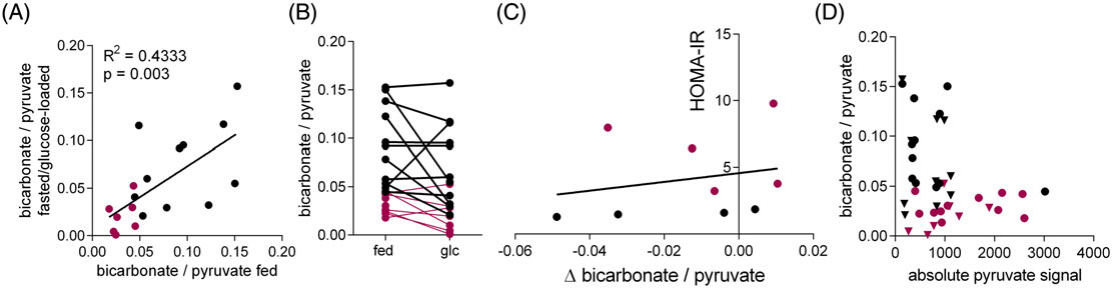
Variability of the myocardial bicarbonate to pyruvate ratio following hyperpolarized [1‐^13^C] pyruvate injection. A, Correlation of bicarbonate to pyruvate ratio in the fed and in the fasted/glucose‐loaded (glc) state. B, Aligned plot indicating each individual rat's bicarbonate to pyruvate ratio progression from the fed to the fasted/glucose‐loaded state. C, Correlation of change (Δ) in bicarbonate to pyruvate ratio in fasted/glucose‐loaded rats compared with the ratio in fed rats and the HOMA‐IR score. D, Correlation of absolute pyruvate signal and bicarbonate to pyruvate ratio of the sum of the first 30 s of spectra. Black circles and triangles indicate control fed and fasted/glucose‐loaded rats, respectively, and magenta circles and triangles indicate T2DM fed and fasted/glucose‐loaded rats, respectively

We next assessed whether individual variability between rats was the driving factor for the overall variability of the bicarbonate to pyruvate ratio or whether intra‐rat differences between fed and fasted/glucose‐loaded rats were also a source of variability. We found a significant correlation of moderate strength between fed and fasted/glucose‐loaded bicarbonate to pyruvate ratios in matched datasets from the same rats (Figure 4A). However, plotting the individual fed versus glucose‐loaded values, it is apparent that there was a degree of variation between different rats, with some rats showing greater bicarbonate to pyruvate ratios after a glucose load and some rats showing the exact opposite (Figure 4B). These changes in bicarbonate to pyruvate ratio between metabolic states (fed and fasted/glucose loaded) were irrespective of the individual rat's insulin sensitivity (Figure 4C). To determine whether the variability in the bicarbonate to pyruvate ratio was due to differences in pyruvate polarization or pyruvate levels in the ventricles, we correlated absolute pyruvate signal in summed spectra from 30 s of data starting from the first appearance of pyruvate to the bicarbonate to pyruvate ratio, and found no correlation between the two measurements (Figure 4D).

**Figure 5:**
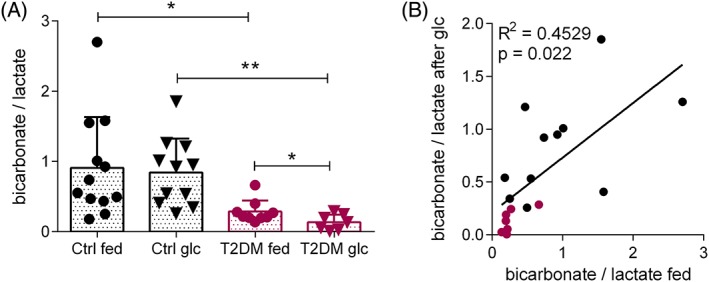
Variability of the myocardial bicarbonate to lactate ratio following hyperpolarized [1‐^13^C] pyruvate injection. A, Bicarbonate to lactate ratio of the sum of the first 30 s of spectra from appearance of the pyruvate peak in fed and fasted/glucose‐loaded (glc) control (Ctrl) and T2DM rats (ctrl fed *n* = 12, ctrl glc *n* = 11, T2DM fed *n* = 9, T2DM glc *n* = 7). Data are expressed as mean ± standard deviation. B, Correlation of bicarbonate to lactate ratio in the fed and fasted/glucose‐loaded states. Black circles indicate control rats and magenta circles indicate T2DM rats. *p < 0.05, **p < 0.01

Normalizing the bicarbonate signal to the metabolic product [1‐^13^C] lactate showed a similar spread of data as the bicarbonate to pyruvate ratio (Figure 5A). In the healthy control rats, the mean bicarbonate to lactate ratio was 0.9 ± 0.2 in the fed state and 0.8 ± 0.1 in the fasted/glucose‐loaded state. In the T2DM rats, the bicarbonate to lactate ratio was higher in the fed state (0.29 ± 0.05) than in the fasted/glucose‐loaded state (0.13 ± 0.04), and this difference was statistically significant (*p* = 0.03). The variance between fed and fasted/glucose‐loaded rats was not different for either control (*p* = 0.73) or T2DM rats (*p* = 0.94). The bicarbonate to lactate ratios in fed rats and after fasting/glucose loading were correlated, but this was less significant than the bicarbonate to pyruvate ratio in both metabolic states (Figure 5B).

**Figure 6:**
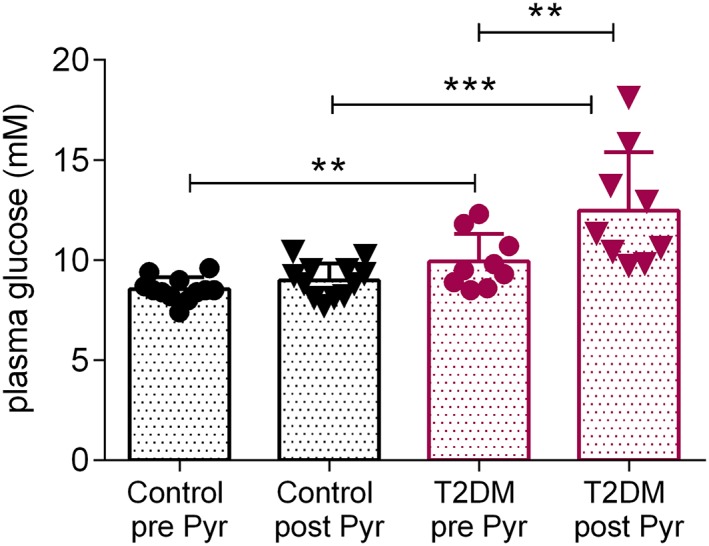
Blood glucose levels in the fed state before and after hyperpolarized [1‐^13^C] pyruvate injection. Blood glucose levels immediately before and 5 min after an intravenous injection of hyperpolarized [1‐^13^C] pyruvate in the fed state. Data are expressed as mean ± standard deviation. **p < 0.01, ***p < 0.001

We finally wanted to assess whether glucose loading after an overnight fast affected the pathologically decreased PDH activity in T2DM hearts, as assessed here through the bicarbonate to pyruvate ratio. Compared with controls, T2DM rats had significantly decreased myocardial bicarbonate labelling from hyperpolarized [1‐^13^C] pyruvate both in the fed state (*p* = 0.0005) and in the fasted/glucose‐loaded state (*p* = 0.0049) (Figure 3C). As for the bicarbonate to pyruvate ratio, the bicarbonate to lactate ratio was also significantly different between control and T2DM fed (*p* = 0.021) and control and T2DM fasted/glucose‐loaded rats (*p* = 0.0016). Neither the lactate to pyruvate nor the alanine to pyruvate ratios were different between the control and T2DM groups in either metabolic state (Figure 3D,E). Interestingly, when diabetic rats were injected with hyperpolarized [1‐^13^C] pyruvate in the fed state, their blood glucose levels increased from 10 ± 1 mM to 12 ± 3 mM (Figure 6), and this was statistically significant (*p* = 0.031). Conversely, blood glucose levels in control rats remained at similar levels (8.6 ± 0.6 mM before and 9.0 ± 0.9 mM after pyruvate injection, *p* = 0.16).

## 4 DISCUSSION

Lactate labelling from hyperpolarized [1‐^13^C] pyruvate in tumour models has previously been shown to be dependent on the level and activity of the lactate dehydrogenase enzyme, total lactate pool size and cofactor (NADH) levels.^34^, ^35^ Similarly, levels of active PDH enzyme have been shown to correlate with PDH flux measurements using hyperpolarized [1‐^13^C]pyruvate,^16^, ^36^ demonstrating the validity of such imaging to determine enzyme flux in vivo. In this study we wanted to determine whether glucose loading after an overnight fast could reduce the observed variability in the myocardial bicarbonate to pyruvate ratio from hyperpolarized [1‐^13^C] pyruvate, which we used as a marker of PDH flux. Glucose infusion had little effect on the inter‐rat variability observed in the myocardial bicarbonate to pyruvate ratio (Figure 4). To exclude the possibility that different pyruvate polarization levels or differences in perfusion or ventricular size leading to different pyruvate levels were responsible for the variability, we correlated the absolute pyruvate levels and bicarbonate to pyruvate ratios in 30 s summed data from the first appearance of the pyruvate peak. We found no correlation between absolute pyruvate signal and the bicarbonate to pyruvate ratio, indicating that bicarbonate to pyruvate ratio variability is independent of polarization level and pyruvate delivery.

Normalizing the bicarbonate signal to lactate instead of pyruvate yielded similar results, further suggesting that variations in injected pyruvate concentration and polarization are unlikely to be a major contributing factor to variability. However, in T2DM rats the bicarbonate to lactate ratio was significantly lower (*p* = 0.03) in the fasted/glucose‐loaded state than in the fed state. As the lactate to pyruvate ratio in these fasted/glucose‐loaded T2DM rats appeared higher than in the fed state, albeit not significantly so (Figure 3D), the differences in the bicarbonate to lactate ratio may be predominantly driven by an increase in lactate labelling. Inter‐individual (i.e. biological) variation probably plays some part in the variability, although the correlation seen between glucose‐loaded and fed ratios within individuals was not supportive of this being the sole factor responsible for the observed variability (Figure 4A,B). The variation between metabolic states (fed compared with fasted/glucose‐loaded) was not dependent on insulin sensitivity (Figure 4C) and therefore must be due to blood‐glucose‐independent mechanisms. For example, levels of circulating FAs may modulate PDH flux.^4^ Isoflurane anaesthesia, as used in this study, has also been shown to attenuate mitochondrial oxidative phosphorylation and glycolytic flux,^37^, ^38^ which modulates cardiac PDH flux detectable by hyperpolarized [1‐^13^C] pyruvate MRS.^39^ We used a constant supply of 2% isoflurane in medical oxygen throughout all our experiments, although there may be variable sensitivity to the anaesthetic gas in our cohort, especially if the rats have different amounts of fat deposits.

The second question we wanted to answer in this study was whether standardized glucose injections would mask the blunted PDH flux previously observed in T2DM rats. We found that fasting/glucose loading did not change the low bicarbonate to pyruvate ratio observed in T2DM rat hearts and that this ratio was significantly lower than in control rats regardless of metabolic state. As an interesting side note we found that hyperpolarized [1‐^13^C] pyruvate injection into fed T2DM rats led to a significantly increased blood glucose concentration after around 5 min. This could be due to increased gluconeogenesis in the liver with pyruvate acting as a gluconeogenic precursor and subsequent export of glucose into the blood stream.^40^ It is also interesting to note that, while glucose concentrations showed a greater spread in the T2DM cohort, the myocardial bicarbonate to pyruvate ratio in these animals was tighter as compared with the control rats, suggesting that maximum achievable PDH flux in the heart is independent of blood glucose levels.

Overall our work suggests that preclinical cardiac hyperpolarized magnetic resonance studies could be performed either in the fed state or after a standardized glucose infusion following and overnight fast. Since PDH flux depends on the physiological state, the availability of substrates and the degree of insulin sensitivity, which may vary in patients with disease, clinical measurements of PDH flux with hyperpolarized [1‐^13^C] pyruvate are expected to be highly variable. Metabolic standardization for hyperpolarized studies in man is currently attempted with an oral glucose load after an overnight fast.^10^, ^11^ Moving from the fasted to the fed state after a fixed glucose load is thought to maximize physiological PDH flux from a standardized (fasted) minimum baseline, thus minimizing variability, although we did not find evidence for this in rats in this study. Centres planning to start new clinical trials with cardiac hyperpolarized magnetic resonance in man may therefore find it beneficial to run small proof‐of‐concept trials to determine whether metabolic standardizations by oral or intravenous glucose load are truly beneficial compared with scanning patients, both with and without diabetes, in the fed state.

### 4.1 Limitations

The main limitation of our study is the use of intravenous glucose loading instead of oral glucose loading as performed in clinical DNP studies.^10^, ^11^ Intravenous glucose loading increases blood glucose levels faster and bypasses the incretin response from the GI tract, which modulates insulin secretion.^41^, ^42^ In patients with diabetes, the incretin response from the GI tract is lower,^43^ which could potentially lead to different effects on hyperpolarized [1‐^13^C] pyruvate metabolism in the heart. While oral glucose loading is desirable in rats, it may be impractical due to animal welfare concerns, especially in longitudinal studies.

## 5 CONCLUSION

In conclusion, we found that making hyperpolarized [1‐^13^C] pyruvate measurements of bicarbonate to pyruvate ratio in either control or T2DM rats in both the fed and fasted/glucose‐loaded states leads to substantial variability in the data with no significant differences between protocols. This variability does not, however, impact on the detection of the diabetic phenotype. While variability did not change between the two different preparations (fed or fasted/glucose loaded), the group means for bicarbonate to pyruvate ratio were slightly altered (albeit not statistically significant), which further strengthens the argument that clear study protocols have to be followed in order to minimize variability and maximize effect sizes between control and disease groups.

## References

[1] Weiss RG , Gerstenblith G , Bottomley PA . ATP flux through creatine kinase in the normal, stressed, and failing human heart. Proc Natl Acad Sci U S A. 2005;102(3):808‐813. 10.1073/pnas.0408962102 15647364 PMC545546

[2] Lopaschuk GD , Ussher JR , Folmes CDL , Jaswal JS , Stanley WC . Myocardial fatty acid metabolism in health and disease. Physiol Rev. 2010;90(1):207‐258. 10.1152/physrev.00015.2009 20086077

[3] Bing RJ , Siegel A , Ungar I , Gilbert M . Metabolism of the human heart II: studies on fat, ketone and amino acid metabolism. Am J Med. 1954;16(4):504‐515. 10.1016/0002-9343(54)90365-4 13148192

[4] Randle PJ , Garland PB , Hales CN , Newsholme EA . The glucose fatty‐acid cycle. Its role in insulin sensitivity and the metabolic disturbances of diabetes mellitus. Lancet. 1963;1(7285):785‐789. http://www.ncbi.nlm.nih.gov/pubmed/13990765 13990765 10.1016/s0140-6736(63)91500-9

[5] Hutson NJ , Kerbey AL , Randle PJ , Sugden PH . Regulation of pyruvate dehydrogenase by insulin action. Prog Clin Biol Res. 1979;31:707‐719. http://www.ncbi.nlm.nih.gov/entrez/query.fcgi?cmd=Retrieve&db=PubMed&dopt=Citation&list_uids=231784 231784

[6] Roche TE , Hiromasa Y . Pyruvate dehydrogenase kinase regulatory mechanisms and inhibition in treating diabetes, heart ischemia, and cancer. Cell Mol Life Sci. 2007;64(7/8):830‐849. 10.1007/s00018-007-6380-z 17310282 PMC11136253

[7] Heather LC , Pates KM , Atherton HJ , et al. Differential translocation of the fatty acid transporter, FAT/CD36, and the glucose transporter, GLUT4, coordinates changes in cardiac substrate metabolism during ischemia and reperfusion. Circ Heart Fail. 2013;6(5):1058‐1066. 10.1161/CIRCHEARTFAILURE.112.000342 23940308

[8] Taegtmeyer H , Young ME , Lopaschuk GD , et al. Assessing cardiac metabolism: a scientific statement from the American Heart Association. Circ Res. 2016;118(10):1659‐1701. 10.1161/RES.0000000000000097 27012580 PMC5130157

[9] Ardenkjaer‐Larsen JH , Fridlund B , Gram A , et al. Increase in signal‐to‐noise ratio of >10,000 times in liquid‐state NMR. Proc Natl Acad Sci U S A. 2003;100(18):10158‐10163. 10.1073/pnas.1733835100 12930897 PMC193532

[10] Cunningham CH , Lau JY , Chen AP , et al. Hyperpolarized ^13^C metabolic MRI of the human heart: initial experience. Circ Res. 2016;119(11):1177‐1182. 10.1161/CIRCRESAHA.116.309769 27635086 PMC5102279

[11] Tyler D , Rider O , Dodd M , et al. Demonstrating the Randle cycle in vivo: assessment of physiological alterations in human cardiac metabolism using hyperpolarised 13C MR spectroscopy. Paper presented at: ISMRM 25th Annual Meeting & Exhibition; April 26, 2017; Honolulu, HI; 726.

[12] Merritt ME , Harrison C , Storey C , Jeffrey FM , Sherry AD , Malloy CR . Hyperpolarized ^13^C allows a direct measure of flux through a single enzyme‐catalyzed step by NMR. Proc Natl Acad Sci U S A. 2007;104(50):19773‐19777. 10.1073/pnas.0706235104 18056642 PMC2148374

[13] Dodd MS , Ball V , Bray R , et al. In vivo mouse cardiac hyperpolarized magnetic resonance spectroscopy. J Cardiovasc Magn Reson. 2013;15(1):19 10.1186/1532-429X-15-19 23414451 PMC3599631

[14] Schroeder MA , Cochlin LE , Heather LC , Clarke K , Radda GK , Tyler DJ . In vivo assessment of pyruvate dehydrogenase flux in the heart using hyperpolarized carbon‐13 magnetic resonance. Proc Natl Acad Sci U S A. 2008;105(33):12051‐12056. 10.1073/pnas.0805953105 18689683 PMC2515222

[15] Golman K , Petersson JS , Magnusson P , et al. Cardiac metabolism measured noninvasively by hyperpolarized ^13^C MRI. Magn Reson Med. 2008;59(5):1005‐1013. 10.1002/mrm.21460 18429038

[16] Atherton HJ , Schroeder MA , Dodd MS , et al. Validation of the *in vivo* assessment of pyruvate dehydrogenase activity using hyperpolarised ^13^C MRS. NMR Biomed. 2011;24(2):201‐208. 10.1002/nbm.1573 20799252 PMC4604661

[17] Lauritzen MH , Laustsen C , Butt SA , et al. Enhancing the [^13^C] bicarbonate signal in cardiac hyperpolarized [1‐^13^C] pyruvate MRS studies by infusion of glucose, insulin and potassium. NMR Biomed. 2013;26(11):1496‐1500. 10.1002/nbm.2982 23794521

[18] Hansen ESS , Tougaard RS , Nørlinger TS , et al. Imaging porcine cardiac substrate selection modulations by glucose, insulin and potassium intervention: a hyperpolarized [1‐^13^C] pyruvate study. NMR Biomed. 2017;30(6):e3702 10.1002/nbm.3702 28186677

[19] Tougaard RS , Hansen ESS , Laustsen C , et al. Acute hypertensive stress imaged by cardiac hyperpolarized [1‐^13^C] pyruvate magnetic resonance. Magn Reson Med. 2018 10.1002/mrm.27164 29524236

[20] Le Page LM , Ball DR , Ball V , et al. Simultaneous in vivo assessment of cardiac and hepatic metabolism in the diabetic rat using hyperpolarized MRS. NMR Biomed. 2016;29(12):1759‐1767. 10.1002/nbm.3656 27779334 PMC5132204

[21] Le Page LM , Rider OJ , Lewis AJ , et al. Increasing pyruvate dehydrogenase flux as a treatment for diabetic cardiomyopathy: a combined ^13^C hyperpolarized magnetic resonance and echocardiography study. Diabetes. 2015;64(8):2735‐2743. 10.2337/db14-1560 25795215 PMC4516266

[22] Rohm M , Savic D , Ball V , et al. Cardiac dysfunction and metabolic inflexibility in a mouse model of diabetes without dyslipidaemia. Diabetes. 2018 10.2337/db17-1195 29610263

[23] Mansor LS , Sousa Fialho M d L , Yea G , et al. Inhibition of sarcolemmal FAT/CD36 by sulfo‐N‐succinimidyl oleate rapidly corrects metabolism and restores function in the diabetic heart following hypoxia/reoxygenation. Cardiovasc Res. 2017;113(7):737‐748. 10.1093/cvr/cvx045 28419197 PMC5437367

[24] Mansor LS , Gonzalez ER , Cole MA , et al. Cardiac metabolism in a new rat model of type 2 diabetes using high‐fat diet with low dose streptozotocin. Cardiovasc Diabetol. 2013;12(1). 10.1186/1475-2840-12-136 PMC3849358 24063408

[25] Morettini M , Faelli E , Perasso L , et al. IVGTT‐based simple assessment of glucose tolerance in the Zucker fatty rat: validation against minimal models. PLoS One. 2017;12(3). 10.1371/journal.pone.0173200 PMC5338807 28264067

[26] Schneider JE , Cassidy PJ , Lygate C , et al. Fast, high‐resolution in vivo cine magnetic resonance imaging in normal and failing mouse hearts on a vertical 11.7 T system. J Magn Reson Imaging. 2003;18(6):691‐701. 10.1002/jmri.10411 14635154

[27] Dodd MS , Atherton HJ , Carr CA , et al. Impaired in vivo mitochondrial Krebs cycle activity after myocardial infarction assessed using hyperpolarized magnetic resonance spectroscopy. Circ Cardiovasc Imaging. 2014;7(6):895‐904. 10.1161/CIRCIMAGING.114.001857 25201905 PMC4450075

[28] Rodgers CT , Robson MD . Coil combination for receive array spectroscopy: are data‐driven methods superior to methods using computed field maps? Magn Reson Med. 2016;75(2):473‐487. 10.1002/mrm.25618 25820303 PMC4744755

[29] Rodgers CT , Robson MD . Receive array magnetic resonance spectroscopy: whitened singular value decomposition (WSVD) gives optimal bayesian solution. Magn Reson Med. 2010;63(4):881‐891. 10.1002/mrm.22230 20373389

[30] Vanhamme L , Van Den Boogaart A , Van Huffel S . Improved method for accurate and efficient quantification of MRS data with use of prior knowledge. J Magn Reson. 1997;129(1):35‐43. 10.1006/jmre.1997.1244 9405214

[31] Bates D , Machler M , Bolker BM , Walker SC . Fitting linear mixed‐effects models using lme4. J Stat Softw. 2015;67(1):1‐48. https://doi.org/10.18637/jss.v067.i01

[32] Kuznetsova A , Brockhoff PB , Christensen RHB . lmerTest Package: tests in linear mixed effects models. J Stat Softw. 2017;82(13):1‐26. https://doi.org/10.18637/jss.v082.i13

[33] Conover WJ , Johnson ME , Johnson MM . A comparative study of tests for homogeneity of variances, with applications to the outer continental shelf bidding data. Technometrics. 1981;23(4):351‐361. 10.1080/00401706.1981.10487680

[34] Day SE , Kettunen MI , Gallagher FA , et al. Detecting tumor response to treatment using hyperpolarized ^13^C magnetic resonance imaging and spectroscopy. Nat Med. 2007;13(11):1382‐1387. 10.1038/nm1650 17965722

[35] Witney TH , Kettunen MI , Day SE , et al. A comparison between radiolabeled fluorodeoxyglucose uptake and hyperpolarized ^13^C‐labeled pyruvate utilization as methods for detecting tumor response to treatment. Neoplasia. 2009;11(6):574‐IN11. 10.1593/neo.09254 19484146 PMC2685446

[36] Dodd MS , Ball DR , Schroeder MA , et al. In vivo alterations in cardiac metabolism and function in the spontaneously hypertensive rat heart. Cardiovasc Res. 2012;95(1):69‐76. 10.1093/cvr/cvs164 22593200 PMC4617603

[37] Kohro S , Hogan QH , Nakae Y , Yamakage M , Bosnjak ZJ . Anesthetic effects on mitochondrial ATP‐sensitive K channel. Anesthesiology. 2001;95(6):1435‐1440. 10.1097/00000542-200112000-00024 11748403

[38] Guerrero‐Orriach JL , Belmonte JJE , Fernandez AR , Aliaga MR , Navarro MR , Mañas JC . Cardioprotection with halogenated gases: how does it occur? Drug Des Dev Ther. 2017;11:837‐849. 10.2147/DDDT.S127916 PMC5358986 28352158

[39] Steinhauser J , Wespi P , Kwiatkowski G , Kozerke S . Assessing the influence of isoflurane anesthesia on cardiac metabolism using hyperpolarized [1‐13C]pyruvate. NMR Biomed. 2017;e3856 10.1002/nbm.3856 29206326

[40] Jin ES , Moreno KX , Wang JX , et al. Metabolism of hyperpolarized [1‐^13^C] pyruvate through alternate pathways in rat liver. NMR Biomed. 2016;29(4):466‐474. 10.1002/nbm.3479 26836042 PMC4805436

[41] Dupre J , Ross SA , Watson D , Brown JC . Stimulation of insulin secretion by gastric inhibitory polypeptide in man. J Clin Endocrinol Metab. 1973;37(5):826‐828. 10.1210/jcem-37-5-826 4749457

[42] Kreymann B , Ghatei MA , Williams G , Bloom SR . Glucagon‐like peptide‐1 7‐36: a physiological incretin in man. Lancet. 1987;330(8571):1300‐1304. 10.1016/S0140-6736(87)91194-9 2890903

[43] Ross SA , Brown JC , Dupre J . Hypersecretion of gastric inhibitory polypeptide following oral glucose in diabetes mellitus. Diabetes. 1977;26(6):525‐529. 10.2337/diab.26.6.525 324834

